# Changes in temperature, pH, and salinity affect the sheltering responses of Caribbean spiny lobsters to chemosensory cues

**DOI:** 10.1038/s41598-019-40832-y

**Published:** 2019-03-13

**Authors:** Erica Ross, Donald Behringer

**Affiliations:** 1 University of Florida, Fisheries and Aquatic Sciences, Gainesville, 32653 USA; 2 University of Florida, Emerging Pathogens Institute, Gainesville, 32608 USA

## Abstract

Florida Bay is home to a network of shallow mud-banks which act as barriers to circulation creating small basins that are often subject to extremes in temperature and salinity. Florida bay is also important juvenile habitat for the Caribbean spiny lobster *Panulirus argus*. While our understanding of the effect of environmental changes on the survival, growth, and movement of spiny lobsters is growing, the effect on their chemosensory abilities has not yet been investigated. Lobsters rely heavily on chemical cues for many biological and ecological activities, and here we report on the effect of extreme environmental events in temperature (32 °C), salinity (45ppt), and pH (7.65 pH) on social behavior and sheltering preference in *P*. *argus*. Under normal conditions, chemical cues from conspecifics are used by spiny lobsters to identify suitable shelter and cues from stone crabs and diseased individuals are used to determine shelters to be avoided. In all altered conditions, lobsters lost the ability to aggregate with conspecifics and avoid stone crabs and diseased conspecifics. Thus, seasonal extreme events, and potentially future climate change conditions, alter the chemosensory-driven behavior of *P*. *argus* and may result in decreased survivorship due to impaired shelter selection or other behaviors.

## Introduction

Coastal ecosystems and the ecosystem services they provide are some of most economically important on the planet. Marshes and mangroves serve as important controls on erosion and pollution^1–3^; near shore reefs and seagrass beds promote nutrient cycling^1–3^; and coastal ecosystems act as important nursery habitats for invertebrate and fish species, many of which support large commercial and recreational fisheries. These ecosystems are therefore some of the most heavily used natural systems and are negatively affected by human activity. Deterioration of coastal ecosystems can lead to increased impacts from biological invasions, fisheries collapse, loss of nursery habitat, decreased water quality, and loss of pollution control. Indirect human induced changes via climate change such as sea level rise (SLR), increasing water temperatures, ocean acidification (OA), and increasing intensity of storm events can also negatively affect coastal ecosystems.

Florida Bay is a shallow lagoonal estuary along the west coast of south Florida and the Florida Keys. It is highly productive, diverse, and provides many distinct, essential, or nursery habitats for a variety of species^4–6^. In fact, 70–90% of all harvested species in the Gulf of Mexico utilize Florida Bay as a habitat for at least one part of their life cycle^7^. The network of shallow mud-banks that are characteristic of this system act as barriers to circulation creating shallow basins that are often subject to extremes in temperature and salinity^4^.

The physical stressors created by the bank system are compounded by human activities such as changes in freshwater delivery from the Everglades and the increasing acidity and temperature associated with climate change. These direct and indirect anthropogenic components have been identified as the dominant stressors affecting key species in the Florida Bay ecosystem^8–10^. Direct human activity through continued alteration in the drainage and flow of the Everglades over the 20^th^ century has decreased freshwater flow into Florida Bay by 59%^11^. Historically, Florida Bay has suffered changes in salinity from freshwater inputs and seasonal droughts, raising salinities up to 70 ppt. These extreme events have been followed by seagrass die offs, algal blooms, and sponge die offs^6,12,13^. Florida Bay also faces seasonal warming spells where temperatures can reach >33 °C^14^. These seasonal extreme events are stressful to marine organisms and are exacerbated by the broader effects of global climate change.

The Caribbean spiny lobster, *Panulirus argus*, is among those species that use Florida Bay as a nursery habitat. *P*. *argus* also supports the single most valuable fishery in Florida and the greater Caribbean, so determining the effects of extreme events on lobster ecology is of utmost importance to coastal communities that depend on it^15–18^. Increasing temperatures and altered salinities are known to affect the survival of spiny lobsters, especially post-larval and juvenile lobsters, which are widespread across Florida Bay^19^. Warming temperatures have also been found to affect other lobsters, such as the European clawed lobster, *Homarus gammarus*, for which warming temperatures were found to shift the timing of larval release by females and negatively affect larval success^20,21^. Low pH significantly decreases growth rates and time to successive molt stage in larvae of the American lobster, *Homarus americanus*^22^, and also decreases the mineral content in the carapace following the final molt stage in *H*. *gammarus*^21^. Although more information like this is emerging about the physiological effects of environmental stressors on lobsters, the effects on their chemosensory ecology is unknown.

The marine environment is rich in chemosensory cues that are important for a wide variety of fundamental biological processes and behaviors. The factors that determine the structure and function of ecosystems are often influenced by chemical cues. Chemical cues determine feeding, habitat, and mate selection^23–25^. Chemical cues are also involved in community dynamics of lower level species, such as algae or bacteria^26,27^. Crustaceans use chemical cues to locate settlement habitat by post-larvae and facilitate prey tracking, predator avoidance, kinship recognition, opponent identification, mate choice, and mating behavior^28–30^. Chemical cues from conspecifics and cohabitants are used by spiny lobsters to form aggregations and identify suitable shelter, while cues from predators, competitors, and diseased individuals are used to determine shelters that are unsafe and should be avoided^31–33^.

Spiny lobsters receive these cues from the environment using setae and sensilla^34,35^. Both olfactory (unimodal) and distributed (bimodal) chemosensilla are found on antennules^35^. The aesthetacs are a specific type of olfactory sensilla, which mediate responses to conspecific cues, including aggregation/attraction cues^36,37^. Chemical cues are delivered to the aesthetacs through rapid flicking of the antennules^38–40^. This flicking motion allows for discrete sampling of the chemical environment. Antennule flicking has been used in a number of studies as an indication of chemosensory ability, as this sensory behavior is responsible for bringing receptor cells in contact with chemical cues^41^.

The effect of temperature on chemoreceptors or chemosensory cues is not currently understood. Increased temperature has corresponding physiological changes on lobsters, which may alter chemosensory motor driven responses, such as walking speeds in search behavior and antennule flicking. Chemoreceptors are also sensitive to salinity. Freshwater has commonly been used in chemosensory studies to inactivate chemoreceptor neurons on the antennules through osmotic shock^29,42,43^. Freshwater destroys the dendritic portion of the chemosensory cells that it contacts but does not kill the cells^43^. Loss of chemoreception is seen for the first day, and sensitivity is slowly regained^42^. The effect of high salinity (>35 ppt) on chemoreceptors in spiny lobsters has not yet been tested, however high salinity (50 ppt for two hours) was used by Kraus-Epley *et al*.^44^ to lesion olfactory appendages of the rusty crayfish, *Orconectes rusticus*^44^. Therefore, seasonal high salinity events in Florida Bay may also trigger osmotic shock similar to fresh water ablations causing short term loss in chemoreceptor function.

Ocean pH levels predicted for 2100 (pH < 7.9) have not yet been reached and pH changes have not been monitored during extreme events in Florida Bay. However, daily fluctuations in ocean pH due to the tidal cycle have been documented. Slight variation in pH has been documented in coral reef ecosystems and benthic stations (5 m) in the Gulf of Mexico were documented to have extreme changes (pH range: 8.05–7.14) in pH throughout the sampling period^45^.

The effect of acidification on growth, reproduction, and fitness of marine animals has been previously documented. More recently the chemosensory abilities of many fish species and other crustaceans have been shown to be affected by CO_2_ enriched environments^41,46–51^, and laboratory experiments with animals in low pH for longer periods translated to field results of animals at CO_2_ seeps^52^. Fish in CO_2_ enriched environments lost the ability to distinguish between unsuitable habitat cues and avoid predators^46,48^. Deep sea hermit crabs in low pH environments (experimental pH = 7.1, ambient pH in the deep sea = 7.6) suffered from impaired olfactory abilities, including prey detection and antennule flicking^41^. Most recently, Roggatz *et al*.^51^ showed that peptide signaling cues of the European shore crab, *Carcinus maenus*, were subject to protonation in future pH conditions (pH = 7.7), which resulted in altered responses and impaired functionality^51^. Low pH during extreme events may, therefore, impact the capacity of lobsters to find appropriate shelter using conspecific cues, and avoid predators and diseased individuals.

The 2013 International Panel on Climate Change (IPCC) report has identified anthropogenic induced climate change as a high-risk factor for the health of coastal marine ecosystems and the services they provide. These seasonal extreme events afford an opportunity to examine how environmental changes at climate change levels affect ecological process. As such, studying their impact can give important insight into the potential future effects of changes in temperature, salinity, and pH.

Here we used the Caribbean spiny lobster as a model to understand how short-duration extreme events may affect well-documented chemosensory-driven attraction and avoidance behaviors and give insight into the possible effects of long-term climate change on these behaviors should they not be able to adapt. Spiny lobsters were used in an experimental choice chamber to test attraction and avoidance responses under different environmental conditions (high temperature, high salinity, high temperature and high salinity, low pH). Attraction behaviors are elicited from healthy conspecifics^31^, and avoidance behaviors are elicited from diseased (Panulirus argus virus 1) conspecifics and stone crabs^32,33^.

## Results

### Response in control conditions

**Figure 1:**
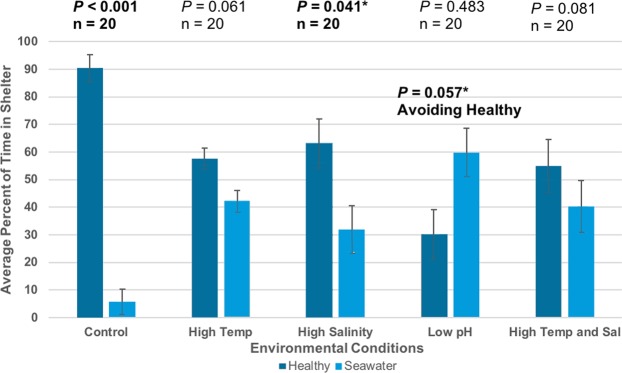
Sheltering choice of focal spiny lobsters in response to healthy conspecific cues. Bars represent average percent of time spent in or within one body length of each shelter. Not all percentages add up to 100%, as these data represent the mean time spent inside a healthy or seawater-only shelter from all trials. Error bars represent 95% confidence intervals. All *p*-values were based on two-tailed binomial tests (α = 0.05). Top *p* values are result of the total time spent in healthy shelters when compared to a null probability of 0.5 or random sheltering. Percent of time in healthy shelters greater than 50% is indicative of attraction behaviors. Additional (*) *p*-value reported for pH treatment is a result of the total time spent in seawater shelter when compared to a null probability of 0.5 random sheltering. Percent of time in seawater shelters greater than 50% is indicative of avoidance behaviors.

**Figure 2:**
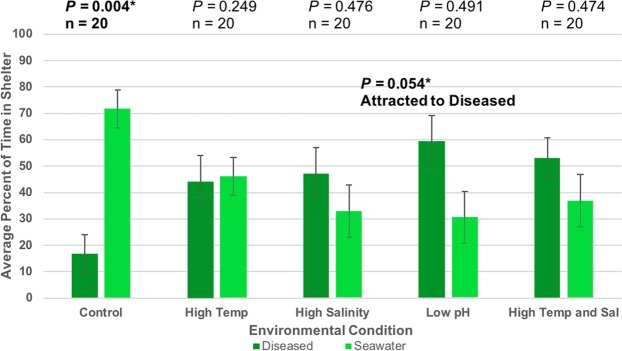
Sheltering choice of focal spiny lobsters in response to diseased conspecific cues. Bars represent the mean percentage of time focal lobsters spent in or within one body length of each shelter. Error bars represent 95% confidence intervals. All *p*-values were based on two-tailed binomial tests (α = 0.05). Top *p*-values are result of the total time spent in seawater shelters when compared to a null probability of 0.5 or random sheltering. Percent of time in seawater shelters greater than 50% is indicative of avoidance behaviors. Additional (*) *p*-value reported for pH treatment is a result of the total time spent in diseased shelter when compared to a null probability of 0.5 random sheltering. Percent of time in diseased shelters greater than 50% is indicative of attraction behaviors.

**Figure 3:**
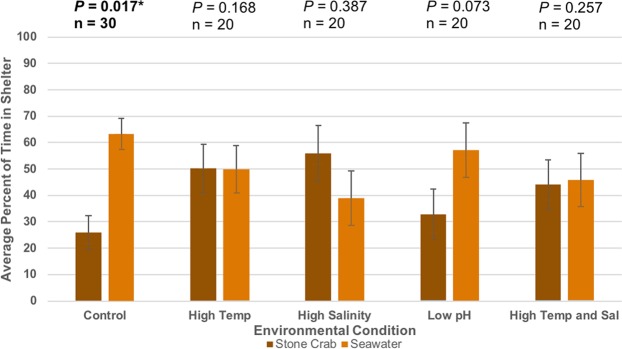
Sheltering choice of focal spiny lobsters in response to stone crab cues. Bars represent the mean percentage of time focal lobsters spent in or within one body length of each shelter. Error bars represent 95% confidence intervals. All *p*-values were based on two-tailed binomial tests (α = 0.05). Top *p*-values are result of the total time spent in seawater shelters when compared to a null probability of 0.5 or random sheltering. Percent of time in seawater shelters greater than 50% is indicative of avoidance behaviors.

Spiny lobsters showed a significant preference for shelters emanating healthy conspecific cues and avoidance of shelters emanating stone crab or diseased conspecific cues. Control trials replicated known attraction and avoidance behavior between conspecifics and stone crabs^31–33^. Lobsters sheltered with healthy conspecific cues significantly more than with seawater-only (90.6% attraction, n = 20, *p* < 0.001) (Fig. 1), and sheltered with seawater-only significantly over diseased conspecifics (71.8% avoidance, n = 20, *p* = 0.004) (Fig. 2). Control trials demonstrated that healthy lobsters significantly avoided stone crabs (63.3% avoidance, n = 30, *p* = 0.017) (Fig. 3).

### Response in high temperature

Spiny lobsters did not show a significant overall preference for, or avoidance of, shelters emanating any cue over seawater in high temperature conditions. Experimental lobsters no longer sheltered with healthy conspecifics significantly over seawater-only (57.8% attraction, n = 20, *p* = 0.061) (Fig. 1), suggesting a loss of attraction behavior at high temperatures. They also no longer sheltered with seawater-only significantly over diseased conspecifics (46.0% avoidance, n = 20, *p* = 0.249) (Fig. 2) or stone crabs (49.8% avoidance, n = 20, *p* = 0.168) (Fig. 3), indicating a loss of both avoidance behaviors.

### Response in high salinity

Experimental lobsters maintained typical sheltering behavior choosing healthy conspecifics significantly over seawater-only in high salinity conditions (63.8% attraction, n = 20, *p* = 0.041) (Fig. 1). Spiny lobsters did not show a significant overall preference for, or avoidance of, shelters emanating diseased conspecific (32.9% avoidance, n = 20, *p* = 0.476) (Fig. 2) nor stone crabs cues over seawater-only (39.1% avoidance, n = 20, *p* = 0.387) (Fig. 3), indicating a loss of both avoidance behaviors in high salinity conditions.

### Response in low pH conditions

Spiny lobsters did not display typical attraction behavior towards shelters emanating conspecific cues (30.2% attraction, n = 20, *p* = 0.483) (Fig. 1). On the contrary, they showed a shelter preference *for* seawater-only shelters that was of borderline significance (59.8% avoidance, n = 20, *p* = 0.057) (Fig. 1). Spiny lobsters did not display typical avoidance behavior in response to shelters emanating diseased cues (30.4% avoidance, n = 20, *p* = 0.491) (Fig. 2). On the contrary, spiny lobsters trended toward a preference *for* shelters emanating diseased cues (59.6% attraction, n = 20, *p* = 0.054) (Fig. 2), although this result was of borderline significance. Spiny lobsters did not show significant avoidance or attraction towards shelters emanating stone crabs cues (57.1% avoidance, n = 20, *p* = 0.073) (Fig. 3), indicating a loss of avoidance behavior.

### Response in high temperature and salinity conditions

Spiny lobsters did not show a significant overall preference for, or avoidance of, shelters emanating cues over seawater-only. Experimental lobsters no longer sheltered with healthy conspecifics significantly over seawater-only (55.0% attraction, n = 20, *p* = 0.081) (Fig. 1), suggesting a loss of attraction behavior in high temperature and salinity conditions. They also no longer sheltered with seawater-only significantly over diseased conspecifics (37.0% avoidance, n = 20, *p* = 0.474) (Fig. 2) or stone crabs (45.9% avoidance, n = 20, *p* = 0.257) (Fig. 3), indicating a loss of both avoidance behaviors.

### Final shelter choice

**Figure 4:**
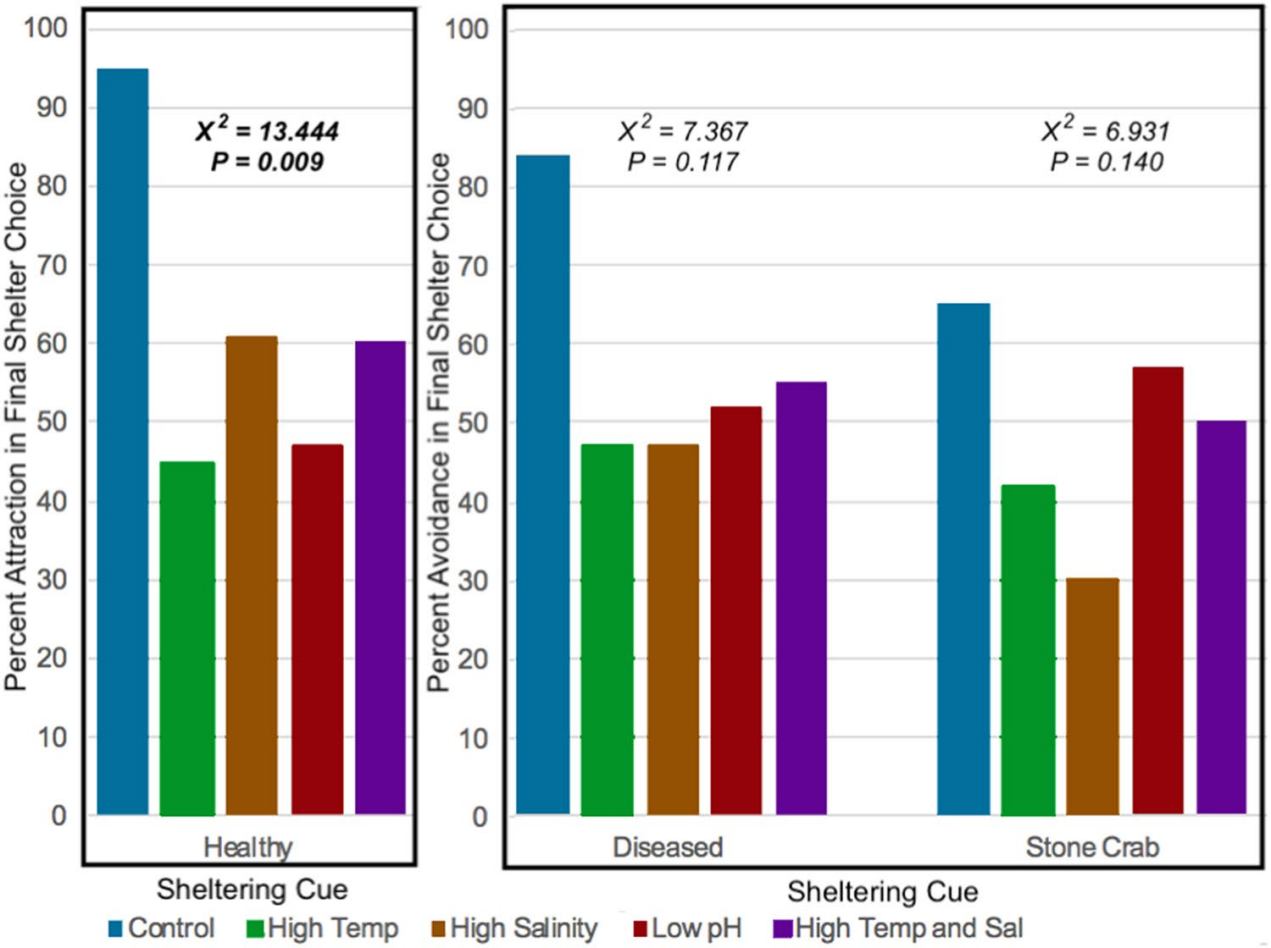
Final shelter choice of spiny lobsters to all sheltering cues. Healthy bars indicate the percent of total trials where the final shelter choice of a focal lobster was with a healthy conspecific over seawater-only. Diseased and stone crab bars indicate the percent of total trials where a focal lobster selected a shelter with seawater-only over a shelter with diseased or stone crab cues, respectively. Choosing a shelter eminating a cue was interpreted as an attraction response (healthy bars) and choosing the other shelter contain seawater-only was interpreted as avoidance (diseased and stone crab bars). Contingency table analysis contrasting shelter choice and all five environmental treatments yielded significantly different preference for healthy conspecific cues between environmental conditions (χ^2^ = 13.444, df = 4, *p* = 0.009). Final shelter choices were not significantly different under altered environmental conditions for diseased conspecific cues (χ^2^ = 7.367, df = 4, *p* = 0.117) or stone crabs (χ^2^ = 6.931, df = 4, *p* = 0.140) across the five environmental treatments.

Control trials showed clear and expected trends in final shelter preference in response to healthy conspecifics, disease conspecifics, and stone crabs (Fig. 4). However, there was no clear shelter preference in any of the altered environmental conditions. Contingency table analysis contrasting shelter choice and all five environmental treatments yielded significant sheltering preference for conspecific cues (χ^2^ = 13.444, df = 4, *p* = 0.009). There was no significant final shelter choice for diseased conspecific cues (χ^2^ = 7.367, df = 4, *p* = 0.117) or stone crabs (χ^2^ = 6.931, df = 4, *p* = 0.140) across the five environmental treatments.

### Antennule flicking

**Figure 5:**
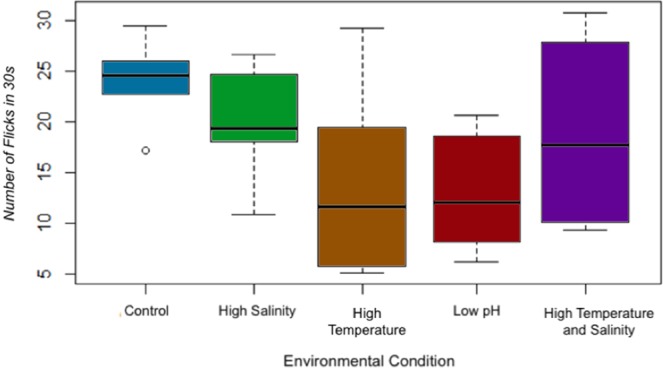
Number of flicks in 30 s in all environmental conditions. Lines in the middle of each box represent the median number of flicks in 30 s for each environmental condition. Removing the outlier in the control group did not change the resulting output, therefore it was left in the analysis. Data represents the composite of all measurements (antennule side, day, and individual; n = 84), as there was no significant effect of day, antennule side or interaction effects in the repeated measures ANOVA. Pairwise comparison of between subject effects showed a trend toward significant effect of environmental condition on antennal flicking rates (df = 4, F = 2.727, *p* = 0.053).

A repeated measures ANOVA with a Huynh Feldt correction found no significant effect of day (df = 4.9, F = 3.381, *p* = 0.107), antennule side (df = 1, F = 4.955, *p* = 0.136), interactions of day with environmental condition (df = 19.674, F = 1.411, *p* = 0.118), interaction of side with environmental condition (df = 4, F = 0.741, *p* = 0.573), interaction of day and side (df = 5.23, F = 0.707, *p* = 0.625) or interaction of day, side and environmental condition (df = 20.935, F = 1.55, *p* = 0.303) in antennule flicking rate. Pairwise comparison of between subject effects did not show any significant difference between antennule flicking rates in altered environmental conditions (df = 4, F = 2.727, *p* = 0.053). However, because the between subject effects were of borderline significance and there were no significant effects of any individual factor (antennule side, day, and individual) a mean was taken of all measurements (antennule side, day and individual) for each environmental condition to assess any overall differences (Fig. 5). Rate of antennule flicking was highest in the control treatment (24.09 flicks in 30 s), followed by the high salinity treatment (19.82 flicks in 30 s), high temperature and high salinity treatment (18.93 flicks in 30 s), high temperature treatment (13.83 flicks in 30 s), and lowest in the low pH group (12.99 flicks in 30 s) (Fig. 5). Control trials show flicking rates consistent (1 Hz) with previous studies on *P*. *argus*^38^.

### Movement Assay

A one-way ANOVA comparing percent of stationary time (time spent not moving), in each treatment and cue combination revealed no significant difference in locomotion behaviors or activity between any treatment or cue combination (df = 14, F = 0.59, *p* = 0.870). Therefore, it is unlikely that general locomotive function or activity was impaired by the environmental treatments.

## Discussion

The coastal ecosystem of Florida Bay is used as a nursery habitat by a large number of ecologically and commercially important species, including the Caribbean spiny lobster. However, its shallow waters are subject to large seasonal changes in temperature and salinity. Human induced climate change resulting in ocean acidification (OA) and an increasing intensity in storm events can also negatively affect coastal ecosystems. Deterioration of coastal ecosystems can lead to increased risk of biological invasions and potentially fishery collapse. Florida Bay is at high risk from seasonal extreme events due to its characteristically shallow, mud-bank system, which is often subject to broad swings in environmental conditions^4–6^.

In this study, we demonstrated that environment perturbations to temperature, salinity, and pH have a strong effect on the chemosensory-driven sheltering behavior of Caribbean spiny lobsters. Control trials supported the attraction behaviors previously observed between healthy conspecifics^31^, and the avoidance behaviors previously reported in response to diseased conspecifics^32,53^ and stone crabs^33^. This confirmed that our bioassay was adequate to determine sheltering preference over a short time period. Changes in temperature, salinity and pH resulted in a reduction of all chemosensory-driven sheltering behaviors tested.

Spiny lobsters did not demonstrate typical attraction or avoidance behaviors in the low pH treatment. Similar to many fish and invertebrate species, we found that lobsters appear to lose chemosensory-driven sheltering abilities when exposed to decreased pH. Similarly, Devine *et al*.^49^ found that adult cardinal fish were no longer able to distinguish between conspecific shelters or locate appropriate shelters after returning from foraging at pH levels of 7.86^49^. Low pH conditions (experimental pH = 4.6, field/collection pH = 5.6) reduced responsiveness of crayfish species to food stimuli^54^. Kim *et al*.^41^ found that chemosensory-driven behaviors of deep-sea hermit crabs were impaired in low pH conditions (experimental pH = 7.1, ambient pH in the deep sea = 7.6)^41^. Crabs which were slowly acclimated to a lower pH over a week were found to have significantly reduced antennule flicking rates and were slower to detect prey. Dixson *et al*.^47^ found that larval clownfish were strongly attracted to predator cues, and no longer able to distinguish between predator and non-predator at pH levels of 7.8 (ambient pH = 8.1)^47^. Here, we show a similar trend towards the reversal of conspecific aggregation and disease avoidance with pH changes from 8.1 to 7.65.

Lobsters were also no longer able to choose suitable shelters in the high temperature treatment, that is, they no longer aggregated with conspecifics or avoided diseased conspecifics or stone crabs. High temperatures were shown to decrease the antennule flicking rate by >50% in our study, although this is a reduction of borderline significance, reduced flicking rates would certainly reduce the ability to detect chemical stimuli, as the flicking rate and speed allow odor molecules to be transported to sensory cells^39,40^. The reduction in antennule flicking rate measured in our study is not likely the sole factor in reduced responses, but it could play some part. To determine how chemoreceptors and chemosensory abilities are specifically affected by an increase in temperature, additional studies should examine the kinematics of the flick and return stroke, and the electrophysiological response from neurons.

Further, spiny lobsters did not demonstrate typical avoidance behavior in response to either diseased conspecifics nor stone crabs in high salinity conditions. Previous work with crustaceans suggests that salinity can play a large role in chemosensory driven behaviors. Kraus-Epley *et al*.^44^ used a two-hour soak in 50 ppt seawater to lesion the olfactory appendages of the freshwater rusty crayfish. Although, they did not confirm that high salinity destroyed the dendritic membranes of chemoreceptors directly, they produced similar bioassay results to this study, confirming significant alteration of orientation behaviors to an odor source at high salinities. These results may indicate that increased salinity is sufficient to dampen the response of lobsters to avoidance cues, as all avoidance behaviors in control trials were significant, but showed more variability when compared to attraction behaviors, and therefore may be more susceptible to change as they are less robust.

The only treatment in which spiny lobsters continued to demonstrate typical sheltering behavior was towards conspecifics in high salinity conditions. Freshwater is commonly used to functionally inactivate chemoreceptor neurons through osmotic shock^43^. Increased salinity or an increase in salinity from 35 ppt to 45 ppt may not be sufficient to completely inactivate chemoreceptor neurons, as the salinity gradient is not as strong as that associated with freshwater knockouts. Rather than a complete loss of function high salinity in our experiment may have partially reduced the availability of functional chemoreceptor neurons, or alternatively, all chemoreceptor neurons may remain intact but suffer from reduced activity in altered conditions. Gleeson *et al*.^43^ found that blue crab *Callinectes sapidus*, acclimated to freshwater, showed significantly reduced olfactory responses when compared to crabs acclimated to saltwater even with all chemoreceptors still functioning^43^. The high salinity in our study may have created a similarly challenging osmotic environment which reduced functionality without completely inactivating chemoreceptor neurons. This may explain the reduced time spent in healthy shelters in high salinity conditions (63%) compared to the time spent in healthy shelters in control conditions (90%). These results suggest that it may take lobsters longer to detect conspecific shelters in high salinity conditions but due to the strong nature of this relationship, they are still attracted to healthy conspecific cues. Although attraction behaviors were maintained in the high salinity that we tested, extreme events in Florida Bay often reach salinities much higher (>50 ppt), potentially creating a stronger osmotic gradient and triggering complete loss of chemoreceptor function^6,12^.

Spiny lobsters did not demonstrate attraction or avoidance behaviors in the combined high temperature and salinity treatment. Loss of attraction and avoidance behavior was likely due to the unknown effect of temperature on chemoreceptors combined with previously documented reduction in functionality of chemoreceptors at high salinities.

Chemosensory-driven sheltering behavior was also measured by final shelter choice, or the location of the lobster at the end of the trial. The final shelter choice in response to healthy conspecifics was consistent with the ‘time spent in a shelter’ results discussed above, showing significantly different shelter preferences under altered environmental conditions. However, the final shelter choice in response to diseased conspecifics and stone crabs were not significantly different under altered environmental conditions. This is likely because even under control conditions, lobster avoidance responses are generally more variable than their conspecific aggregation responses. Therefore, time spent within shelters was a better indicator of actual shelter preference than the final shelter choice.

Reduced chemosensory ability, but not complete loss of function, may result in insufficient cue recognition to ensure shelter choice, so the animal leaves the shelter and samples the cue eluting from the other side. These results could also indicate that the animal was under duress and was seeking to move out of the adverse conditions, regardless of the chemosensory information received. However, this is unlikely the case in our study, as no animals died during the course of the experiment, animals continued normal eating behavior and results from the movement assay showed lobsters in altered environmental conditions did not move more or less than when under control conditions. Decreased time near shelters could also indicate a loss or impairment of visual ability, but that was not measured in this study.

Regardless of the effect on chemoreceptors, the overall results showed a reduction in all classic shelter preferences in conditions that occur during the typical extreme event in Florida Bay. Loss of chemosensory-driven disease avoidance could be devastating for juvenile lobsters. Juveniles first settle into the shallow waters of Florida Bay as pueruli, where the highest increase in water temperatures and salinity occur with seasonal extreme events. As small juveniles, they are also at a much higher risk of infection by PaV1 than larger lobsters^55,56^. Avoidance behavior has significant advantages in reducing the risk of infection and disease transmission in wild populations^32,57^. Therefore, disruption of their normal pathogen avoidance behavior puts juveniles inhibiting Florida Bay at higher risk of contracting and spreading PaV1. Changes in disease avoidance can have significant effects on the spread of the PaV1 virus, and attraction to diseased conspecifics in low pH conditions would likely have detrimental effects considering avoidance of infected lobsters appears to reduce the potential for drastic increases in prevalence^13^.

Future studies are needed that focus on the function of the chemoreceptors to unequivocally document that environmental changes result in impaired chemosensory ability versus impaired motor function. Here we document changes in chemosensory-driven behaviors, but the source of this change in behavior is not explicit. Reduction in motor responses, such as flicking, which enhance chemosensory detection by the antennules may also be involved. Changes in cue structure, which prevent detection by receptors, or decreased in the efficiency of receptor binding could also alter chemosensory responses^51,58^. Roggatz *et al*.^51^ found that peptide mediated behaviors of *Carcinus maenus* were functionally impaired in low pH conditions through changes in peptide cue structure and electrostatic properties, which altered receptor binding^51^. Chemical cues used in head tanks were collected from source animals held at constant, normal conditions and then the water containing the cue was altered to match the environmental treatment of interest. This was done because we do not know how environmental change might affect the normal release of attraction and avoidance cues (e.g., the release of urine) and we sought to ensure that the cues were released as normal. This could have altered cue structure and prevented detection. However, the exact structure, and bioactive molecules of the conspecific aggregation cue, PaV1 disease avoidance cue, and stone crab avoidance cue of *P*. *argus* are currently unknown. The identification and characterization of these bioactive molecules may further illuminate the root cause for changes in behavior that we observe. Although this study does not unequivocally demonstrate that chemosensory function was compromised, the effect is the same – lobsters did not respond to avoidance or attraction chemosensory cues as they normally would.

In conclusion, the lack of avoidance of diseased individuals and attraction to suitable shelters may have stark consequences for spiny lobster populations and crustaceans more broadly. These environmental conditions are seen seasonally in Florida Bay, and loss of conspecific aggregation may decrease the effectiveness of the trap fishery, which relies on the gregarious nature of *P*. *argus* to attract other lobsters to the trap. Furthermore, the seasonal loss of avoidance behavior in response to conspecifics infected with PaV1 could increase the prevalence of disease and the loss of avoidance response to stone crabs could result in increased lobster injuries that decrease growth rates and increase their susceptibility to disease^59^. Reduced survival due to impaired shelter selection under altered environmental conditions would have negative consequences for spiny lobster populations already impacted by reduced habitat quality and overfishing. These effects may be more prominent for crustaceans living in shallow nearshore areas where extreme events are more pronounced and frequent. These results may also serve as a portend for the chemosensory-driven changes we might expect under projected climate change scenarios. Global CO_2_ emissions are continuing to rise as a result of anthropogenic climate change, further increasing ocean temperatures, decreasing pH, and in some coastal areas, increasing salinity. Further, seasonal droughts coupled with raising temperatures often result in increased incidences of extreme weather events, and even higher salinities due to decreased freshwater flow. Here, we show that all of these changes can affect the response of spiny lobsters to ecologically important chemosensory cues.

## Methods

### Animal husbandry

Juvenile *P*. *argus* (n = 160, carapace length [CL] 50–60 mm) were collected via hand-net from hard-bottom habitat in the Florida Keys (USA) and held in three UV-treated recirculating tanks (600 L) under a natural photoperiod for 1-week prior to the experiment. Juvenile lobsters (n = 10, 50–60 mm CL) used to provide attraction cues were housed separately from experimental lobsters in 190 L tanks. Diseased *P*. *argus* (n = 5, 30–40 mm CL) were identified as those that were visibly infected with PaV1 (white hemolymph)^55^, and were housed separately in a 190 L tank. The number of diseased individuals was limited due to the inability to readily find visibly diseased individuals who will survive longer than a few days. Further, we used a smaller size class of diseased lobsters because PaV1 is highly infectious to smaller juveniles and it is very rare to obtain a sufficient number of larger PaV1 positive individuals^55,56^. The stone crab, *Menippe mercenaria*, is a common inhabitant of the coastal waters of Florida Bay and was chosen because it is an aggressive competitor that spiny lobsters have been documented to avoid using chemical cues^33^. Stone crabs (n = 10, 80–90 mm carapace width [CW]) were housed separately in a two 190 L tanks (5 each).

### Experimental Design

**Figure 6:**
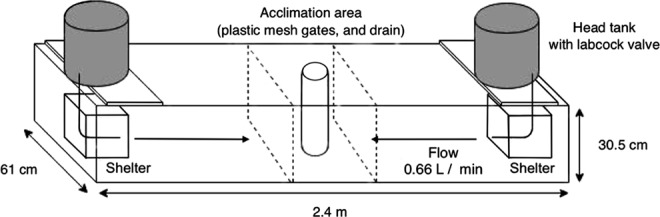
Experimental choice chamber and set up for behavioral assay A cinderblock shelter was placed at each end. The head tanks (seawater-only, cue treatment) rested on shelves above the choice chamber. Head tank cue treatments were created by incubating one animal providing the desired cue in the head tank under control conditions for 24 h. Prior to the start of the trail, the head tank water was adjusted to match the environmental condition of the choice chamber. Flow rate from each end of the choice chamber was 0.66 L min^−1^, and drained through a central PVC stand pipe with holes symmetrically placed around the pipe.

The experimental choice chambers (2.4 × 0.6 × 0.3 m, 152 L; Fig. 6) consisted of a standpipe drain in the center, a central acclimation area, and a cinderblock shelter at each end. Head tanks (20 L) were placed above each end of the chamber, and a silicon tube delivered seawater (with or without a chemical cue) from the tank into the center of a shelter in the chamber beneath it. Flow rate (gravity-fed; 0.66 L min^−1^ at the beginning of each trial) from each head tank was controlled using a plastic ball valve and measured daily. Dye tests confirmed that flow was unidirectional and the rate was equal from both ends of the choice chamber. All trials were performed under ambient photoperiod, during daylight hours, which prompts the nocturnal lobsters to seek shelter. All experiments were recorded remotely using a Q-See 4 Channel IP Bullet Camera digital security system to ensure that lobsters were not affected by observation. Video analysis was done blind with respect to cue and seawater-only side.

Each environmental variable treatment (control, high temperature, high salinity, high temperature and high salinity, low pH) used a unique set of lobsters (n = 20 per treatment), and no lobster was used across treatment groups. The control trials for stone crab were increased to 30 trials as there is only one study indicating this response, and it was under review during our experimentation^33^. Each experimental lobster for a specific treatment group was tested with all cue scenarios (healthy conspecifics, diseased conspecifics, stone crab) as collecting and housing enough unique animals for each cue scenario was not feasible and responses to different cues were independent. No lobsters were used more than once per day, no positive or negative conditioning stimulus was provided, and the order of cue exposures was randomized to eliminate any effects of learning from using an animal under multiple cue scenarios.

All lobsters to be used in a specific environmental treatment were housed together in a 600L recirculating tank and habituated to that environmental condition slowly over 7d and any adjustments to the water were made in the tank sump, to ensure thorough mixing. They were then held at those conditions until all trials were run (~10d). Temperature was measured in the holding tank and increased 1–2 °C each day for 7d using a submersible Finnex aquarium heater placed in the tank sump. Salinity was measured in the main holding tank and increased 2 ppt each day for 7d via slow addition of Instant Ocean^TM^ artificial seawater to the tank sump. The pH was lowered with the addition of CO_2_ via a Green Leaf Aquarium^TM^ CO_2_ system controlled by a Milwaukee pH controller in the main holding tank, which injected CO_2_ gas through a diffuser into the tank sump. The set pH was lowered by 0.05 each day for 7d until it reached 7.65, where it was held for the remainder of the experiment by the CO_2_ system controller. In the low pH treatment, the temperature (25 ± 1 °C), salinity (35 ± 3 ppt), and pH (7.65 ± 0.05) were monitored in the holding tank throughout the day and adjusted as necessary via the sump water.

To ensure that animals providing chemical cues were not inhibited from doing so by altered environmental conditions, the head tank cue treatments were created by incubating one animal providing the desired cue in the head tank under control (temperature 25 ± 1 °C, salinity 35 ± 3 ppt, pH 8.1 ± 0.05) conditions for 24 h. Prior to the start of the trail, the head tank water was adjusted to match the environmental condition of the choice chamber so that it mixed readily when the head tank water flowed in. This step was essential for diseased lobsters, which may not have survived altered conditions. The animals used for attraction and avoidance cues were fed the day prior to the 24 h incubation period in the head tank.

All behavioral trials were conducted in choice chambers that were separate from the holding tanks and in freshly mixed artificial seawater of the same environmental condition in which animals were habituated. The treatment conditions in the choice chambers were created and maintained in the same method as the habituation tanks. Seawater was made 24 h prior to any behavioral trials were run to ensure that all treatment conditions were stable.

### Experimental manipulations

An experimental lobster was allowed to habituate for 5 min in the central acclimation area of the choice chamber^36^. The side of the chamber from which the chemical cue (or seawater-only) was released was determined using a random number generator. Preliminary tests (seawater vs seawater) showed that there was no significant side preference in the choice chamber. Rhodamine dye tests showed that the 5 min habituation period was sufficient time for the odor plume to reach the holding area and central drain. Preliminary trials also showed that 5 min was sufficient time for the experimental lobster to regain typical resting behavior. The gates between the holding area and working area of the choice chamber was then lifted, allowing the lobster to move freely. Each trial proceeded for 30 min, as preliminary trials demonstrated that this was sufficient time to observe typical shelter use, attraction, or avoidance behavior. Further, as described in Horner *et al*.^36^, placing the animal in natural flow conditions allows for a more rapid laboratory assay^36^. At the conclusion of the trial, the chamber was drained, rinsed with freshwater, and dried to ensure no chemical cues remained for the next trial.

High temperature treatments (32 ± 1 °C) were determined from field measurements of seasonal warming events, and NOAA monitoring stations located throughout Florida Bay (Vaca Key SST, mean 32 °C 2005–2012). High salinity treatments (45 ± 3 ppt) were based on field measurements of seasonal extreme events and historical data^6^. Baseline pH conditions were determined from the NOAA monitoring buoy at Cheeca Rocks (mean pH 8.11 2012). Low pH trials were run with a pH of 7.65 ± 0.05, the projected ocean pH by 2100 as there are no current pH measurements from extreme events in Florida Bay^21,22,60,61^. A high temperature and salinity treatment was also included, which tested increased salinity and temperature in tandem since these conditions are apt to occur simultaneously in seasonal extreme events. Environmental conditions in the choice chamber were measured at the beginning of each trial to ensure the treatment level fell within the desired range noted above.

### Chemosensory ability (antennule flicking)

The rate of antennule flicking was used to determine chemosensory ability as flicking is equivalent to ‘sniffing’ in many crustacean species^38–40^. The rate of antennule flicking was recorded for each antennule (right and left side) on six individuals for seven consecutive days as the number of flicks in 30 s. Each animal was held individually in a 40 L glass aquaria. A unique set of six animals was used in each environmental condition. Flicking was measured for each environmental treatment (control, high temperature, high salinity, high temperature and high salinity, and low pH). All animals were habituated to the environmental condition slowly over 7d as above. Video was recorded from the anterior position of each lobster so both antennules were clearly visible. No stimulus to elicit a flicking response was provided, and all measurements were taken at the same time of day in full light conditions.

### Data analysis

The sheltering behavior of lobsters was measured by analyzing the recordings for total time spent in each shelter, and final shelter choice (location at the end of the 30 min trial). Total time spent in each shelter was determined as the time the experimental lobster was in or within one body length (“near”) from the shelter. When near the shelter the sides of the shelter offered partial protection, and the lobsters were still able to sample water emanating from the shelter. Although these lobsters near the shelter did not enter the shelter, they were using the shelter for safety so were included in total time spent within each shelter. These sheltering measurements were also used by Horner *et al*.^36^.

The time spent in (or near) each shelter was standardized by converting it to a percentage by dividing it by the total time spent in (or near) both shelters combined. This excluded the amount of time spent outside of one body length from a shelter. This percentage was compared to 50%, or no shelter preference, using two-tailed binomial tests (α = 0.05). Previous studies have analyzed sheltering preference in this manner^32,53,62,63^ and to yield results comparable to these studies we elected to use this convention. Furthermore, using the null hypothesis, or a random 50/50 selection, was the most conservative estimate for comparing behavioral changes. Final shelter choice was categorical data, so independence between treatments was determined using a *X*^2^ contingency table analysis.

A repeated measures ANOVA was used to determine the effect of environmental condition on antennule flicking rates. The data on number of flicks were normally distributed (Shapiro Wilks test, *p* > 0.05). The assumption of homogeneity of variances was violated as assessed by Levene’s test for equality of variances. However, ANOVA tests are relatively robust to violations of this assumption provided group sizes are equal, which they were. Mauchly’s test of sphericity indicated that assumption of sphericity was violated (*p* < 0.05), therefore a Huynh-Feldt correction was applied.

To rule out general malaise or the possible effect of environmental treatments on motor function, a movement assay was also conducted. A one-way ANOVA was used to investigate the percent of ‘stationary time’ (time spent not moving) in each environmental treatment.

## Data Availability

The datasets generated and analyzed during the current study are available from the corresponding author on reasonable request.
